# Intraindividual reaction time variability affects P300 amplitude rather than latency

**DOI:** 10.3389/fnhum.2014.00557

**Published:** 2014-07-29

**Authors:** Anusha Ramchurn, Jan W. de Fockert, Luke Mason, Stephen Darling, David Bunce

**Affiliations:** ^1^Department of Psychology, Goldsmiths, University of LondonLondon, Greater London, UK; ^2^Division of Psychology and Sociology, Queen Margaret UniversityEdinburgh, UK; ^3^Faculty of Medicine and Health, Institute of Psychological Sciences, University of LeedsLeeds, UK

**Keywords:** P300, intraindividual variability, ERP, reaction time, executive function

## Abstract

The neural correlates of intraindividual response variability were investigated in a serial choice reaction time (CRT) task. Reaction times (RTs) from the faster and slower portions of the RT distribution for the task were separately aggregated and associated P300 event-related potentials computed. Independent behavioral measures of executive function and IQ were also recorded. Across frontal, fronto-central, central, centro-parietal and parietal scalp regions, P300 amplitudes were significantly greater for faster relative to slower behavioral responses. However, P300 peak amplitude latencies did not differ according to the speed of the behavioral RT. Importantly, controlling for select independent measures of executive function attenuated shared variance in P300 amplitude for faster and slower trials. The findings suggest that P300 amplitude rather than latency is associated with the speed of behavioral RTs, and the possibility that fluctuations in executive control underlie variability in speeded responding.

The possibility that trial-to-trial reaction time (RT) variability may provide a behavioral marker of central nervous system integrity has generated considerable interest. For example, behavioral research shows that increased intraindividual RT variability (IIV) is associated with traumatic brain injury (e.g., Stuss et al., 1994), epilepsy (Bruhn and Parsons, 1977) and mild dementia (e.g., Hultsch et al., 2000). There is also work showing that greater IIV is associated with attention deficit hyperactivity disorder (e.g., Klein et al., 2006), older age (e.g., Hultsch et al., 2002; Bunce et al., 2004; Lövdén et al., 2007), mild psychopathology (Bunce et al., 2008a,b), and impending mortality (Macdonald et al., 2008a). Additionally, age differences in within-person variability extend to real-world activities such as driving performance (Bunce et al., 2012).

By contrast, considerably less research has investigated the brain correlates of IIV and the principal aim of the present research was to help address this shortfall. Existing work includes several MRI studies that have shown a relationship between IIV and specific brain structures. For example, structural MRI studies reveal an association with microscopic white matter lesions in the frontal cortex (Bunce et al., 2007, 2010), white matter connectivity (Deary et al., 2006) and corpus callosum thickness (Anstey et al., 2007), while fMRI work shows systematic IIV-related activity in the dorsolateral prefrontal (Bellgrove et al., 2004) and parietal cortices (MacDonald et al., 2008b). Indeed, work also implicates involvement of striatal dopamine D2 receptor binding (MacDonald et al., 2009), a finding that is consistent with the idea that IIV may reflect neural noise in the brain (Li et al., 2001). Although these imaging studies clearly suggest brain structures or activity to be associated with IIV, a limitation is that they provide little insight into the temporal synchronicity between brain processes and behavior. It is important, therefore, to demonstrate a systematic association between within-person variability in behavioral response latencies and neural activity.

Although little research has investigated the neural correlates of RT inconsistency using electrophysiological techniques, existing evidence provides some important clues. For example, there is an association between the endogenous P300 ERP component and IIV in persons who have experienced head trauma (Segalowitz et al., 1997), and recent work has confirmed a systematic association between that component and the degree of within-person variability (Saville et al., 2011). The P300 component is a positive-going deflection occurring approximately 200–400 ms following stimulus onset. Also referred to as P3b or “classic P3” elicited by repetitive stimuli, it is distinguished from the P3a or “novelty P3 potential” elicited by infrequent stimuli (Knight et al., 1989). Delineating these sub-components according to topographical scalp distribution, P3a generally shows maximum amplitude at fronto-central sites (Comerchero and Polich, 1998, 1999; Simons et al., 2001), while the consensus based on young adults is that P3b is more parietally distributed and shows maximal amplitude over the parietal/central area (Herrmann and Knight, 2001). Previous research (see Coles and Rugg, 1995) suggests the P300 reflects stimulus evaluation time and context, or in some tasks, memory updating. Importantly, the P300 is also held to index processes involved in working memory (Donchin et al., 1986), and reflects demands placed upon attentional resources (e.g., Polich, 1987; Kramer and Strayer, 1988).

The electrophysiological studies investigating IIV have predominately used either a between-participant design, where persons exhibiting high behavioral IIV are contrasted with persons of low variability, or correlational methods where behavioral variability measures are regressed on EEG metrics. Since the early work of Morrell and Morrell (1966) and Donchin and Lindsley (1966), to our knowledge no research has used a within-subject design where ERP components elicited by a visual task have been delineated for behavioral RTs from the faster and slower reaches of the distribution. As such an analysis may provide important insights into the cognitive operations that underlie IIV, it is this research shortfall that motivates the first major objective of the present study. Evidence suggests that increased IIV may arise from a greater proportion of slower responses as opposed to a general slowing of RTs across the entire distribution (e.g., Rabbitt, 1989; Rabbitt and Maylor, 1991; Spieler et al., 2000; West et al., 2002). This raises the question of whether there is a qualitative shift in the cognitive operations underlying the two types of response. Such a shift may reflect differences in the type, depth, or efficiency of information processing, and may suggest that the greater proportion of intermittently slower responses contributing to increased RT inconsistency may arise from attentional lapses (Bunce et al., 1993) or relatedly, fluctuations in executive control (West et al., 2002; Bunce et al., 2004). Central to these suggestions is the idea that an executive supervisory system varies in its efficiency and behaviorally these fluctuations manifest as within-person RT variability.

Our approach to IIV is similar to the between-participant method in that it measures the variation in an individual’s responses in terms of latency. What is novel in our approach is that in order to identify any factors that are shared between responses from different portions of the response distribution, we have binned together trials on the basis of latency and compared the electrophysiological correlates associated with the different latency bins. IIV is determined by temporal variation in an individual’s RTs across a task and in particular, differences between RTs falling into the faster and slower reaches of the individual RT distribution. A key question is whether the cognitive and neural processes underpinning an individual’s faster RTs differ from those underpinning the slower RTs. If differences are detected, it may help shed light on the role of executive and attentional control mechanisms in IIV. Moreover, investigating IIV by comparing responses from the same task conditions between faster and slower sections of the RT distribution makes it possible to examine variability within the same individual. Importantly, it is likely that the factors that drive between-participant differences in variability as reflected by a person’s standard deviation, including noise at perceptual and response stages of processing, lapses of attention, and fluctuations in cognitive load, are likely to be the same as those tapped into by comparing faster with slower latency bins.

Our first objective in the present study was to investigate P300s obtained from EEG activity associated with faster and slower responding in a visual 2-choice RT task. By comparing ERPs from RTs adjacent to the center of the distribution with those from the slower end of the distribution, we sought to delineate the neural activity, and by implication, the cognitive operations associated with the two types of response. Given the association with attentional engagement and executive processes described earlier, our main interest was in the amplitude of the P300 ERP component. In addition, following suggestions that faster and slower RTs are related to fluctuations in attentional or executive control, our second objective was to record independent behavioral measures of those constructs. We reasoned that, if such measures are supported by similar executive processes to those implicated in RT inconsistency, they would statistically account for the variation in the brain activity associated with faster and slower responding.

## Method

### Participants

Behavioral and electrophysiological data were recorded for 16 right-handed participants aged 21–33 years (*M* = 25.48, *SD* = 3.19). All participants were university graduates (6 women), recording a mean predicted full-scale IQ (National Adult Reading Test: NART; Nelson, 1982) score of 108.88 (*SD* = 8.74). Participants were free from performance-affecting medications. Informed consent was obtained prior to the study, which had received appropriate ethics approval from the local research ethics committee.

### ERP behavioral task

A 2-choice reaction time (CRT) task was administered via a PC time-locked to the EEG recording equipment. Participants sat at a viewing distance of 70 cm from the screen. A white central fixation-cross appeared for 850 ms, the onset of which was jittered by either 250, 500, or 750 ms. This was followed by a 2000 ms blank pre-stimulus interval screen. Stimuli (a white 1 cm diameter circle) were then randomly presented 75 mm either left or right of the fixation-cross for 200 ms, followed by a 2500 ms blank response screen. Participants used their left or right index finger to indicate the location of the circle by pressing the appropriate one of two keys mounted on a response box. Instructions emphasized speed and accuracy, and that participants should attempt to keep their eyes fixated on the central cross (to minimize eye movements).

Twenty-four practice trials followed by six blocks of 50 test trials were administered. This procedure allowed participants frequent breaks to minimize fatigue. The length of pause between blocks was participant determined. Following each block of trials, participants were asked to rate how demanding the block they had just completed was (1 = Not at all demanding; 10 = Extremely demanding). Statistical comparisons of demand scores across blocks did not identify any significant differences (*p* > 0.26).

Preparation of data for behavioral and ERP analyses was as follows. Error trials were eliminated (1.23% of trials), as were trials where response latencies fell below 150 ms (1.31% of trials), the minimum threshold for valid responses suggested by prior research (Hultsch et al., 2002). It was also desirable to remove abnormally slow responses exceeding the individual’s mean RT by 3 SDs (1.17% of trials). As these procedures removed outliers from both extremes of the distribution and therefore reduce IIV, this represents a conservative approach to the investigation of within-person variability.

### ERP recording and data processing

A 32-channel elasticated cap containing silver/silver chloride electrodes (Quick-Cap, NeuroMedical Supplies, El Paso, Texas) configured to the 10–20 system (Jasper, 1958) was fitted to participants. Vertical and horizontal eye movements were detected via electrodes placed at the outer canthi of both eyes (HEOGL, HEOGR, VEOGU, VEOGL). The ground electrode was placed at midline, approximately 8.5 cm above the eyebrows. Impedances were reduced to below 5 kΩ using abrasion and electrolyte gel (Quick gel, NeuroMedical Supplies, El Paso, Texas). EEG recordings were made using NeuroScan SynAmps running Scan 4.2 software (Neuroscan Compumedics, El Paso, Texas). Signals were recorded with a DC (baseline) to 30 Hz bandpass and amplified with gain of 500 and a sampling rate of 500 Hz. During recording, the experimenter monitored electrode saturation levels using DC correction where appropriate.

Following recording, DC drift and offset corrections were applied to the EEG data. Trials affected by eye movements were automatically rejected using a voltage threshold of ±30 for both vertical (VEOG channel) and horizontal (HEOG channel) movements. For valid trials, ERP epochs were created for each electrode spanning 100 ms prior to, and 450 ms after, stimulus onset.

To compute the electrophysiological correlates of response variability for each individual, P300s were extracted for trials falling into either the second or fourth quartiles of the behavioral RT distribution and aggregated for subsequent analyses. Mean amplitudes (μV) were calculated within a 40 ms measurement window (295–335 ms) centered around the peak of the component. This strategy was adopted as (i) quartiles would include sufficient trials to form bins of a minimum of 20 trials for ERP averaging; and (ii) it was important to assess trials drawn from the faster relative to the slow end of the distribution. Even though a minimum threshold of 150 ms for valid responses was used, trials from the first quartile would still be likely to include instances of extremely fast RTs due to rapid but correct anticipations or guesses. Thus, the second quartile was preferable as it excluded such trials, but included a substantial proportion of the faster trials in the main distribution (i.e., faster than but proximate to the median). We elected to eliminate trials from the third quartile from the analyses as this had the advantage of creating a clear separation between faster and slower RT trials. This optimized the RT difference between conditions (i.e., it excluded portions of the distribution that were neither fast nor slow). Importantly, this strategy allowed us to investigate electrophysiological activity from the slower portion of the RT distribution, identified by prior research to be responsible for increases in intraindividual variability, relative to activity from the faster reaches of the distribution.

### Behavioral measures of executive function

Measures of executive functioning capturing the constructs of switching, updating, and inhibition (Miyake et al., 2000) were administered according to standard protocols. These measures have also been used to assess frontal lobe function (e.g., Parkin et al., 1995; Robertson et al., 1997). Verbal fluency was measured using the FAS test where participants were allowed 1 min to generate as many unrelated words beginning with “F” and then, in turn, “A” and “S” (Benton et al., 1983). Here, the total score for the three letters is used (*M* = 47.44, *SD* = 9.72). Additionally, the Food Test and a task combining verbal fluency and switching (see Parkin et al., 1995) were administered. In the Food Test, participants were given 1 min to generate as many supermarket items of food as possible without repetition, while in the switching task, 1 min was allowed to generate as many alternate Animal-Country name switches as possible without error or repetition. The number of correct responses in these tasks was subjected to statistical analysis (Food Test *M* = 27.25, *SD* = 6.48; Switching *M* = 23.50, *SD* = 5.51). Two tasks were used to measure inhibitory function. First, a PC version of the Stroop color-word task (96 test trials, where half the color-word combinations were congruent, and half incongruent) was administered where participants were required to respond to the ink color of the presented word rather than the color represented by the word (red, yellow, green, blue). Here we report the percentage of errors for incongruent trials (*M* = 3.58, *SD* = 3.44). Second, the Sustained Attention to Response Task (SART) was administered via a PC according to the original specifications for the random sequence version of the task (see Robertson et al., 1997). This task is a Go/No-Go task where single digit numbers were presented on the computer screen. Participants were required to respond to all numbers via the space bar as quickly as possible with the exception of the number “3” for which responses had to be withheld. Here, the percentage of erroneous responses to the number “3” were recorded (*M* = 4.83, *SD* = 3.61). Additionally, a composite measure of executive function was created by subjecting the above variables to principal component analysis and saving the factor scores.

### Procedure

On arrival at the laboratory, participants completed informed consent and a short questionnaire recording biographical information. Measures of executive function were administered and then participants were fitted with caps for the EEG procedure. On completion, participants were debriefed and paid £15 for participation.

## Results

**Figure 1:**
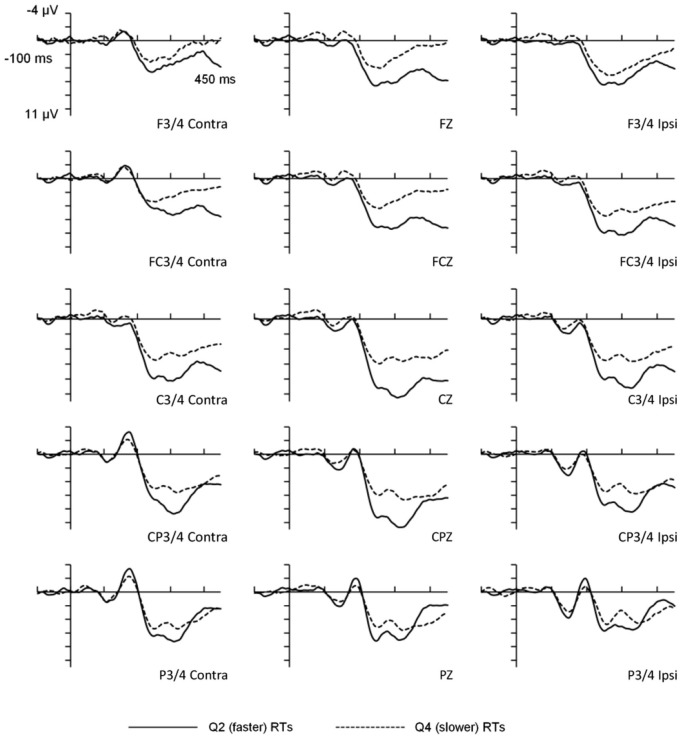
**Waveforms for faster and slower reaction times as a function of response lateralization.** The figure illustrates ANOVA analyses for frontal, fronto-central, central, centro-parietal, and parietal scalp-electrode sites. RT Length refers to faster vs. slower RTs. Contra = activation measured on the opposite side to the visual field to which stimuli were presented. Ipsi = activation measured at the electrode site on the same side as the probe side.

For the behavioral data, mean RTs for the faster (2nd quartile) and slower (4th quartile) portions of the distribution were 401 ms (*SD* = 53 ms) and 630 ms (*SD* = 87 ms) respectively, *T*_(15)_ = 22.94, *p* < 0.001. Importantly, the mean within-participant RT variability also differed significantly between the faster (mean *SD* = 11 ms) and slower (mean *SD* = 114 ms) portions of the distribution, *T*_(15)_ = 6.9, *p* < 0.001. Overall error rate was low (*M* = 1.23%) and error rates were not analyzed. The visual probe stimuli elicited an evoked potential comprised of several positive and negative peaks (see Figure 1), the most prominent being a positive deflection peaking at 315 ms which was maximal at centro-parietal electrode sites. The scalp distribution and timing of this deflection was consistent with the visual P300 component.

### ERP component analysis

Brain activity sampled from frontal (F3/4/z), fronto-central (FC3/4/z), central (C3/4/z), centro-parietal (CP3/4/z), and parietal (P3/4/z) electrode scalp locations formed the first of three within-subjects factors in a 5 (Electrode site) × 3 (Laterality: midline/ipsilateral/contralateral) × 2 (RT length: faster/slower) ANOVA. Mean amplitude for the P300 served as the main dependent variable, and we additionally measured the mean amplitude of the visual visual P1 (122–162 ms) and N1 (170–210 ms) ERP components elicited by the probe stimuli.

The possibility that the probe presentation side may account for the findings was also formally assessed in a separate 5 × 3 × 2 × 2 ANOVA on P300 amplitudes where the additional within-subject factor was Probe-side. However, this did not reveal a significant main effect for Probe-side (left/right visual field) or interactions with our key variable of interest, RT Length (faster/slower). Thus, Probe-side is not considered further. In the interest of determining the specificity of any P300 effects, additional analyses performed on the P1 and N1 at electrode sites P3/4 contralateral, Pz, and P3/4 ipsilateral showed that the main effect of RT Length was not significant for either component, indicating that these early exogenous components exhibited no systematic variation with response time. There was no interaction between quartile length, hemisphere and region.

### P300 amplitude

The main effect for Electrode site was statistically significant, *F*_(4,60)_ = 11.36, *p* < 0.001, *η*^2^ = 0.431, and Bonferroni-adjusted *T*-tests indicated that the centro-parietal region (*M* = 7.36, *SD* = 4.30) recorded greater amplitudes than frontal (*M* = 3.77, *SD* = 5.45), fronto-central (*M* = 4.96, *SD* = 5.17) and parietal (*M* = 5.46, *SD* = 4.06) regions, but not the central region (*M* = 7.040, *SD* = 4.88). The main effect of Laterality was also significant, *F*_(2,30)_ = 8.42, *p* < 0.001, *η*^2^ = 0.359, and further Bonferroni-adjusted *T*-tests identified that amplitudes at electrode locations contralateral to the side of stimulus presentation (*M* = 5.17, *SD* = 4.47) were smaller than those either ipsilateral (*M* = 5.77, *SD* = 4.95) or at midline (*M* = 6.22, *SD* = 5.41). Ipsilateral and midline amplitudes did not significantly differ. These main effects were not modified by a higher-order interaction. Most importantly though, the main effect for RT Length was significant, *F*_(1,15)_ = 21.68, *p* < 0.001, *η*^2^ = 0.591, with larger P300 amplitudes for faster (*M* = 7.20, *SD* = 4.83) than for slower responses (*M* = 4.24, *SD* = 4.67).

This main effect was modified by a statistically significant interaction between Laterality and RT Length, *F*_(2,30)_ = 27.59, *p* < 0.01, *η*^2^ = 0.333, and *post-hoc* one-way ANOVAs assessing Laterality were significant for both faster and slower RTs, *F*_(2,30)_ = 13.66, *p* < 0.001 and *F*_(2,30)_ = 4.68, *p* < 0.05, respectively. Bonferroni-adjusted *T*-tests showed that for faster RTs, P300 amplitudes were greater for the midline (*M* = 7.98, *SD* = 5.37) compared to contralateral (*M* = 6.68, *SD* = 4.43) and ipsilateral (*M* = 6.94, *SD* = 4.56) measures. For slower RTs, amplitudes were greater for the contralateral (*M* = 3.66, *SD* = 3.98) compared to midline (*M* = 4.46, *SD* = 4.87) and ipsilateral (*M* = 4.59, *SD* = 5.07) measures, but only the latter survived a Bonferroni correction. Thus, the P300 for faster responses was more centrally distributed (larger at the midline) than for slower responses.

The interaction between Electrode site and RT Length was also significant, *F*_(4,60)_ = 43.71, *p* < 0.01, *η*^2^ = 0.247, and *post-hoc* one-way ANOVAs assessing Electrode site (F, FC, C, CP, P) within each level of the RT Length factor were significant for both faster, *F*_(4,60)_ = 13.91, and slower RTs, *F*_(4,60)_ = 6.25, both *p*s < 0.001. Bonferroni-adjusted *T*-tests paired relative to the centro-parietal region (where P300 was maximal) identified that for faster RTs, P300 amplitudes were larger at centro-parietal regions (*M* = 9.07, *SD* = 3.99) compared with frontal (*M* = 4.84, *SD* = 4.97), fronto-central (*M* = 6.65, *SD* = 5.20), and parietal (*M* = 6.36, *SD* = 3.75) regions, and that central P300s (*M* = 9.07, *SD* = 3.99) were larger than parietal P300s (*M* = 6.36, *SD* = 3.75). For slower RTs, the two comparisons that survived Bonferroni adjustments suggested that centro-parietal P300s (*M* = 5.66, *SD* = 3.91) were larger than P300s at both parietal (*M* = 4.56, *SD* = 4.18) and fronto-central (*M* = 3.28, *SD* = 4.58) electrode sites. Thus, while faster responding elicited the largest P300s across central and centro-parietal electrode sites with amplitudes differing markedly from other areas, slower responding elicited less distributed activation with maximal P300 amplitude localized over the centro-parietal region. The three-way interaction involving Laterality, Electrode site and RT Length was nonsignificant.

The principal feature of our findings so far is that mean P300 amplitudes for faster trials were significantly greater than those for slower trials. Additionally, the significant interactions of Electrode site and Laterality by RT Length suggests that faster responding was associated with more distributed maximal P300 activation (across central and centro-parietal regions, particularly midline) compared to slower responding, which is concentrated over the centro-parietal region.

### P300 peak amplitude latency

In addition to the association between P300 amplitude and behavioral RT length, it was important to assess whether P300 peak amplitude latency was also associated with behavioral RT latency. This would provide information as to whether the P300 amplitude or timing was primarily associated with behavioral response speed. Additionally, a possible explanation for the observed P300 amplitude reduction as a function of behavioral RT Length was that the effects were due to differences between RT latency conditions in terms of the degree of latency jitter of the P300 component.

These concerns were formally addressed using a jackknifing procedure (Ulrich and Miller, 2001) that increased the signal-to-noise ratio of the ERPs.^1^ Neither the comparison for peak latency (Faster: *M* = 315, Slower: *M* = 317, *p* > 0.91) nor peak latency variance were statistically significant. These findings suggest that it was the amplitude rather than either the amplitude latency or the latency jitter of these components that was primarily associated with behavioral response times.

### Executive control, P300 and IIV

A further objective of the study was to assess the extent to which independent measures of executive function accounted for the differences in mean amplitude associated with faster and slower behavioral responses. To this end, for the electrode sites where P300 amplitudes were maximal (CP3/4/z), we conducted a series of hierarchical multiple regressions where in the initial model, the P300 amplitude from faster RTs was regressed onto the P300 amplitude from slower RTs. In a second model, this procedure was repeated but entering the respective executive function variables at Step 1. The key element of this procedure is the reduction of *R*^2^ change from Model 1 to Model 2 when executive function is taken into account at Step 2. As IQ correlated significantly with some of the ERP components (see Table 1), all of the regression models were adjusted for this variable at Step 1.

**Table 1:** **Bivariate correlations between IQ, executive function variables and P300 amplitudes for faster (quartile 2) and slower (quartile 4) responding at electrode sites where P300 was maximal**.

	**CP3 fast**	**CP3 slow**	**CP4 fast**	**CP4 slow**	**CPz fast**	**CPz slow**
IQ	−0.499*	−0.514*	−0.382	−0.356	−0.440	−0.482	
FAS	0.277	0.352	0.369	0.369	0.315	0.323	
Food	0.086	0.237	0.038	0.238	0.089	0.139	
Switching	0.015	−0.141	0.266	0.409	0.050	−0.048	
Stroop	−0.360	−0.562*	−0.145	−0.605*	−0.481	−0.595*	
SART	−0.377	0.103	−0.223	0.052	−0.304	0.100	
Composite measure	0.340	0.275	0.316	0.482	0.375	0.269	

SART = Sustained Attention to Response Task; * *p* < 0.05.

The results of the regressions are presented in Table 2. As can be seen, in the first model, significant changes in *R*^2^ were obtained for each of the three P300 components. Importantly though, for several of the equations, an attenuation in the change in *R*^2^ was obtained from Model 1 to Model 2 having taken executive function into account, and four of these increments became nonsignificant (CP3/FAS, CP4/FAS/switching/composite measure). For example, for CP3, the initial change in *R*^2^ was 0.30 but when the FAS task was taken into account in Model 2, the change in *R*^2^ reduced to 0.15 and became nonsignificant. Together, these attenuations in *R*^2^ change are consistent with the interpretation that executive function is accounting for some of the shared variance between amplitudes for faster and slower RT trials.

**Table 2:** **Hierarchical multiple regression: P300 amplitude for faster (quartile 2) responses regressed on P300 amplitudes for slower (quartile 4) responses, adjusting for executive function (Model 2)**.

**Executive function variable**	**CP3 fast ∆*R*^2^**	**CP4 fast ∆*R*^2^**	**CPz fast ∆*R*^2^**
*Model 1*			
None*^a^*	0.30*	0.28*	0.41**
*Model 2*			
FAS*^b^*	0.15	0.12	0.24*
Food*^b^*	0.28*	0.28*	0.39**
Switch*^b^*	0.31*	0.20	0.41**
Stroop*^b^*	0.22*	0.36*	0.25*
SART*^b^*	0.34**	0.29*	0.44**
Comp*^b^*	0.19*	0.17	0.28*

All models adjusted for IQ;

Comp = composite measure of executive function, SART = Sustained Attention to Response Task;

a = df = 1,14; b = df = 1,12;

*p < 0.05, **p < 0.01.

## Discussion

This study adds to evidence suggesting that behavioral IIV is systematically related to brain activity. Uniquely, the investigation also provided insights into the association between independent behavioral measures of executive function and that activity. There were several important findings. First, P300 amplitudes were significantly greater for faster relative to slower behavioral responses while peak amplitude latency obtained for faster and slower responding did not differ significantly, either in terms of mean peak latency, or latency jitter. Importantly, these findings suggest that it was amplitude rather than the latency that was associated with variation in behavioral RTs. As P300 amplitude is widely held to index the allocation of attentional resources (e.g., Polich, 1987; Kramer and Strayer, 1988), these findings are of note as they suggest that attentional and executive control play a key role in behavioral response variability.

Second, although the pattern of P300 amplitudes for faster and slower responses was distributed across the scalp, interactions involving Laterality and Region suggested that amplitudes for faster responses were larger across the midline, with a more widely distributed maximal amplitude (i.e., across central and centro-parietal regions). In contrast, P300 for slower responding elicited larger amplitudes at lateralized locations and showed maximal amplitude in only the centro-parietal region. These effects suggest that faster responding is associated with a more widely distributed network of processing and perhaps greater recruitment of attentional resources than slower responding. Finally, several of the independent behavioral measures of executive function attenuated the shared variance between P300 amplitude for faster and slower RTs. This finding is consistent with the view that variation in P300 amplitudes for the respective response speeds was related to the efficiency with which executive control was engaged.

Given evidence that increased IIV may arise from a greater proportion of intermittent slower responses (e.g., Rabbitt, 1989; Rabbitt and Maylor, 1991; Spieler et al., 2000; West et al., 2002), our findings are of particular note as they suggest that although faster and slower behavioral responses were supported by the same cognitive processes, for slower trials, those processes were not fully engaged. Our reasoning here is that whereas the P300 amplitude was significantly greater for the faster responses than for the slower responses, the latency or latency jitter of that component did not differ according to response type. This clearly suggests that differences in behavioral response speed were related to the amplitude rather than the timing of the P300. The opposite pattern, where the P300 varies in latency but not amplitude, would indicate that the longer behavioral RTs were the consequence of similar, but slower, cognitive operations.

Additionally, independent measures of executive function statistically accounted for the differences in P300 amplitudes associated with the faster and slower behavioral responses (FAS, switching, composite measure). Although the effects were modest, the findings suggest that executive resources supporting performance across these tasks may underlie the P300 amplitude differences observed for faster and slower responding. Moreover, as in much previous work investigating the link between executive function and the P300 (e.g., Jackson et al., 1999; Brydges et al., 2014; Samyn et al., 2014), the current evidence for an association is indirect, and further research investigating the possible role of executive function in accounting for RT within-person variability would be worthwhile.

Together these findings point to the possibility that top-down cognitive operations involved in executive control underlie IIV. As such, they support theoretical accounts suggesting that within-person variability in responding may reflect fluctuations in executive control (West et al., 2002; Bunce et al., 2004) or relatedly, attentional lapses (Bunce et al., 1993). Indeed, there is functional imaging work (Bellgrove et al., 2004; MacDonald et al., 2008b, 2009) demonstrating systematic variation in BOLD signal and response variability, and work also suggesting that slower behavioral responses may arise from attentional lapses (Weissman et al., 2006). The P300 has been proposed to index the time taken to evaluate a stimulus (Coles and Rugg, 1995), working memory engagement (Donchin et al., 1986), or the allocation of attentional resources (e.g., Polich, 1987; Kramer and Strayer, 1988). As executive control is central to all of these cognitive operations, it is likely that the variations in P300 amplitude found for faster and slower responses in the present study reflected moment-to-moment fluctuations in engagement of attentional and executive control mechanisms.

Our ERP findings suggest that IIV is associated with systematic differences in the magnitude of certain brain responses, rather than their timing. Although ERP methodology provides excellent temporal resolution in relation to the brain’s electrical activity, the spatial origins of the activity are subject to a certain amount of speculation. However, functional MRI work (Bellgrove et al., 2004) suggests that the dorsolateral prefrontal cortex plays an important role in regulating behavioral response latencies and also that extrastriatal D2 dopamine receptor binding is implicated in IIV (MacDonald et al., 2009). Our findings indicate that IIV effects are unlikely to be limited to the frontal cortex. Given the link between IIV and neurobiological disturbance, it is important that research explores the specific role of various brain structures and processes in IIV further.

There are some potential limitations to the present findings that we should acknowledge. The first concerns the influence of fatigue. It has long been understood (e.g., Broadbent, 1958) that occasionally slower responses across the course of an extended vigil are related to the accumulation of fatigue associated with time-on-task. As it was necessary to eliminate this potential confound, we designed the study such that the 300 trials were administered in blocks of 50, thereby allowing participants to rest between blocks. As subsequent analysis of perceived demands did not reveal any significant differences across block, it appears unlikely that our findings were confounded by block-to-block variation in perceived demands and associated fatigue. Additionally, the impact of time-on-task was formally investigated by contrasting P300 amplitudes for the first and second half of the session. While main effects of time-on-task indicated that the P300 was larger in the second compared to the first half of the task, these effects did not interact with RT Length, suggesting that time-on-task effects did not contribute to the observed P300 amplitude differences for faster and slower responses.

Second, it is possible that trial-to-trial latency jitter may have contributed to the P300 amplitude differences between faster and slower trials. That is, the variability in the timing of peak P300 amplitude from trial-to-trial may have attenuated the amplitudes when aggregated. Although we recognize this to be a potential problem in work of this type, we do not believe this unduly affected the main findings of this study because the same 40 ms “window” was set for both faster and slower P300 latencies (295–335 ms) and therefore the windows were equally constrained. Indeed, consideration of the waveforms presented in Figure 1 shows that the overall temporal profile is very similar for faster and slower responses. This suggests that the amplitude differences are unlikely to be due to timing differences. Furthermore, when P300 peak latency variances for faster and slower RTs were contrasted, the results were not statistically significant, indicating that jitter did not explain the amplitude differences observed. Finally, while it is important to consider issues relating to multicollinearity in using multiple regression procedures such as those for the mediator analyses, variance inflation factors were <2.0 for all of the models with the majority around 1.5 and below. These statistics, therefore, do not suggest multicollinearity to have been a major issue in the regression analyses.

The present findings provide important background information for studies reporting increased RT inconsistency in populations exhibiting neuropathology such as traumatic brain injury (e.g., Stuss et al., 1994), epilepsy (Bruhn and Parsons, 1977), attention deficit hyperactivity disorder (e.g., Klein et al., 2006), mild dementia (e.g., Hultsch et al., 2000), and also in older adults (e.g., Hultsch et al., 2002; Bunce et al., 2004; Lövdén et al., 2007). The study provides further evidence that the neural correlates of faster and slower serial responses differ and suggests that IIV systematically reflects endogenous neural processes (e.g., Hultsch et al., 2008).

To conclude, the study indicates that the amplitude rather than the latency of the P300 is associated with faster and slower behavioral responding. Additionally, as independent measures of executive function attenuated the effect sizes for differences in P300 amplitude associated with faster and slower responses, it is possible that those differences reflect fluctuations in attentional engagement and executive control. As top-down cognitive operations associated with central nervous system integrity may govern IIV, it suggests that behavioral measures of RT inconsistency may have considerable potential as quick-to-administer supplements to existing neuropsychological batteries used in clinical assessment. It is important that future work investigates this potential further.

## Conflict of interest statement

The authors declare that the research was conducted in the absence of any commercial or financial relationships that could be construed as a potential conflict of interest.

## Footnotes

^1^Specifically, we measured both the peak latencies and the variance in latency for each participant and for each RT quartile (Q2 and Q6). A separate average ERP was calculated for each participant *i* (*i* = 1 … *n*) by temporarily omitting participant *i* and computing a peak latency from the averaged data of the remaining *n*-1 participants. A variance score was calculated from these data for each participant and for each RT quartile (Q2 and Q6) and was submitted to a *t*-test with a correction factor of *t*corr = *t*/(*n*-1) (Kiesel et al., 2008).
